# Longitudinal correlation of 3D OCT to detect early stage erosion in bovine enamel

**DOI:** 10.1364/BOE.8.000954

**Published:** 2017-01-18

**Authors:** Abdirahman Aden, Paul Anderson, Gary R. Burnett, Richard J. M. Lynch, Peter H. Tomlins

**Affiliations:** 1 Barts & The London School of Medicine and Dentistry, Institute of Dentistry, Queen Mary University of London, E1 1BB, UK; 2 GSK Consumer Healthcare, St. Georges Avenue, Weybridge, Surrey, KT13 0DE, UK

**Keywords:** (100.0100) Image processing, (120.0120) Instrumentation, measurement, and metrology, (110.4500) Optical coherence tomography, (170.0170) Medical optics and biotechnology, (120.3890) Medical optics instrumentation, (170.1850) Dentistry

## Abstract

Erosive tissue-loss in dental enamel is of significant clinical concern because the net loss of enamel is irreversible, however, initial erosion is reversible. Micro-hardness testing is a standard method for measuring initial erosion, but its invasive nature has led to the investigation of alternative measurement techniques. Optical coherence tomography (OCT) is an attractive alternative because of its ability to non-invasively image three-dimensional volumes. In this study, a four-dimensional OCT system is used to longitudinally measure bovine enamel undergoing a continuous erosive challenge. A new method of analyzing 3D OCT volumes is introduced that compares intensity projections of the specimen surface by calculating the slope of a linear regression line between corresponding pixel intensities and the associated correlation coefficient. The OCT correlation measurements are compared to micro-hardness data and found to exhibit a linear relationship. The results show that this method is a sensitive technique for the investigation of the formation of early stage erosive lesions.

## 1. Introduction

The loss of dental hard tissue is multifactorial, contributed to by abrasion between teeth and other materials, attrition and acid erosion. Dental erosion can be defined as an irreversible loss of dental hard tissue due to a chemical process and without bacterial involvement [1]. However, during the early stages of erosion the tooth surface undergoes a superficial partial dissolution of mineral [2] leaving a partially intact, but softened layer of enamel. The thickness of the softened layer is estimated to be 2-5 μm [3,4]. Detection of erosion at this stage is critical because the remaining softened enamel can serve as a scaffold for new mineral. Prolongation of the challenge may result in further dissolution, ultimately leading to tissue loss [5]. The loss of enamel volume is characterised by a persistent softened layer at the surface of the remaining tissue [6,7]. One of the major causes of erosion is the consumption of acidic drinks and researchers are keen to quantify the amount of erosion that various beverages may cause [8]. Quantification of erosion is necessary to provide further insight into the chemical process of erosion, and ways in which it can be modified, reduced or prevented.

The gold standard for the measurement of early stage surface softening (ESS) is micro-hardness testing [9]. However, this is typically an *in vitro* technique, reliant upon physical indentation of the specimen. Clinically, erosion can be measured on casts obtained from dental impressions [10] using surface profilometry or visually at the chairside using an ordinal scoring system [11]. However, these techniques are only sensitive to bulk mineral loss that is beyond remineralisation. To the authors’ knowledge, ESS measurement using dental X-Rays has not been reported in the literature. Therefore, various techniques have been proposed for detecting, imaging, monitoring and quantifying dental hard tissue mineralisation dynamics, both *in vitro* and *in vivo* [12]. Among these techniques, Optical Coherence Tomography (OCT) has been explored for caries detection [13–19] and to a lesser extent for erosion measurement [20,21]. Notably, ESS has not been widely studied using OCT.

Predominantly, studies have employed two-dimensional OCT B-Scan images to directly monitor lesion formation. For example, *in vivo*, 15.3 μm of erosion was measured over a 3 week period in patients having Gastroesophageal Reflux Disease (GERD) [21]. Enamel thickness was estimated from OCT B-Scans by measuring the distance from the enamel surface to the enamel dentine junction (EDJ). Similarly, enamel thickness was monitored *in vitro* with OCT [22] as a measure of erosive progression. However, the authors reported many challenges with accurately measuring the remaining enamel thickness. They found that if erosion is accompanied by subsurface demineralisation or surface roughness, the strong increase in scattering limits the ability to resolve the EDJ. A well hydrated surface can somewhat mitigate against this by providing a degree of refractive index matching and consequently lower scattering and increased light penetration. However, prior to the net loss of enamel, measurement of ESS remains a challenge for OCT. Detection of ESS using OCT cross-sectional B-Scan images is difficult because intense specular reflections from the enamel surface mask information about scattering within the first few micrometres below the tooth surface [23]. Nevertheless, some progress has been made by measuring changes in the intensity of the backscattered OCT signal, leading to the detection of erosive lesion formation after 10 minutes of continuous acid challenge at pH 3.8 [20].

Consequently, OCT is promising because it provides real-time imaging and does not require specimen processing. Moreover, OCT could be developed into a technique for the *in vivo* characterisation of erosive lesions that is both non-ionising and minimally invasive [24]. With OCT, an entire area of the specimen can be measured non-destructively. This is advantageous because it allows for more accurate averaging over specimen heterogeneity compared to measuring a few discrete indentations as with micro-hardness. Furthermore, OCT can re-measure the same area longitudinally. This is not possible with destructive techniques.

Typically, techniques for *in vitro* longitudinal erosion measurement require specimens to be removed from the acidic solution and dehydrated prior to measurement. Such an approach may disrupt the softened region [25] and introduce variations in sample hydration [26], that in OCT has been shown to reduce sensitivity to demineralisation and therefore contributes to experimental uncertainty. Repositioning accuracy has previously been addressed by placing control markings to aid realignment of successive measurements [20]. Nevertheless, manual repositioning limits the accuracy of longitudinal measurements because successive B-Scans are not guaranteed to be spatially co-registered. This can be a significant limitation where only single B-Scans are acquired for analysis at each time-point [20,27] and becomes increasingly important for detecting small changes such as in ESS [28]. Therefore, the design of the present study aimed to avoid specimen movement and drying thus mitigating against the consequent uncertainties.

The primary aim of this study was to use a 4D OCT system (three orthogonal spatial dimensions and time) to determine whether it could detect ESS and, if so, whether a statistical relationship with hardness measurements could be established.

## 2. Theory

Erosion is predominantly a surface effect, with ESS resulting in microscopic changes to surface structure, composition and mineral density. Three-dimensional OCT datasets contain surface information, which can be visualised in the *en face* plane as opposed to the cross-sectional B-Scan view typical of previous studies. Consequently, under acidic challenge, the optical scattering properties of an enamel surface are expected to change. In OCT, changes to the distribution of optical scatterers can lead to two dominant effects; a change in the scattering phase function and an altered speckle pattern.

The scattering phase function provides a measure of the angular dependence of the intensity of scattered light. Thus, changes to it necessarily affect both the intensity of light coupled from the surface into the OCT detection optics and the intensity of light that scatters forward. Consequently, surface changes are expected to yield a change in the intensity of sub-surface light coupled back into the OCT system. Assuming that a polished specular surface has optimal transmission characteristics, then disruption to this through ESS might be expected to decrease the sub-surface OCT signal, i.e. making OCT B-Scan images appear less bright. Therefore, detection sensitivity to small surface changes would be enhanced by integrating the OCT signal over the full depth to which light penetrates into the specimen. Such a projection of the sample can be approximated by integrating the OCT linear intensity volume *I*(*x*,*y*,*z*) over some axial range *z*_1_ to *z*_2_ within which the specimen surface and sub-surface structures of interest are located. Mathematically this is expressed as Eq. (1),

$$P(x,y)=\int_{z_{1}}^{z_{2}}I(x,y,z)\text{d}z$$ (1)

where *x* and *y* represent orthogonal lateral dimensions of the *en face* view and *z* the axial direction into the sample.

Speckle is the visual manifestation of coherent interference between light scattered from closely spaced scattering centers. These effects have previously been exploited in optical coherence elastography (OCE) whereby an applied stress displaces scatterers and consequently modifies the speckle pattern [29]. Furthermore, optical coherence angiography (OCA) is used to image microvasculature [30]. Intensity based OCA relies upon changes to the speckle pattern in blood vessels with respect to the surrounding tissue resulting from the movement of red blood cells. Although a number of methods exist for parameterising speckle and intensity changes, one attractive method is the measurement of statistical correlation [31] because its magnitude is always scaled between 0 and 1. Specifically, Pearson’s product moment correlation coefficient, *r*, is a measure of the linear relationship between two variables, for example *P*_0_(*x*,*y*) and *P*_t_(*x*,*y*), representing two projection intensities located at *x* and *y* obtained at times 0 and *t* minutes. In this case, the correlation coefficient can be calculated from Eq. (2) as,

$$r(t)=\frac{\sum_{x}\sum_{y}\left[P_{0}(x,y)-\bar{P_{0}}\right]\left[P_{t}(x,y)-\bar{P_{t}}\right]}{\sqrt{\sum_{x}\sum_{y}{\left[P_{0}(x,y)-\bar{P_{0}}\right]}^{2}{\sum_{x}\sum_{y}\left[P_{t}(x,y)-\bar{P_{t}}\right]}^{2}}}$$ (2)

where the horizontal bar represents the mean of the quantity beneath it. Then, *r*, is a measure of the strength and direction of the linear relationship between intensities in the two images. OCT images are discretely sampled in *x*, *y* and *z.* Therefore, the projection images comprise discrete pixels.

**Fig. 1:**
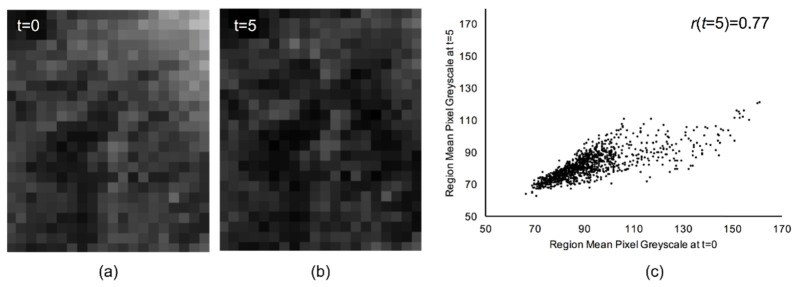
Example of spatially registered projection images at acid challenge durations of a) *t* = 0 minutes and b) *t* = 5 minutes expanded to show individual pixels. c) The intensity of spatially corresponding pixels, plotted such that intensities from *t* = 0 are along the horizontal axis and intensities from *t* = 5 are on the vertical axis.

For the sake of demonstrating the principles of correlation analysis, examples of enamel projection images are shown in Fig. 1(a)

and Fig. 1(b) corresponding to *P*_0_(*x*,*y*) and *P_t_*_= 5_(*x*,*y*). These represent an enamel surface before and after a 5 minute acid challenge. The relationship between pixel intensity values at *t* = 0 minutes and *t* = 5 minutes are shown in the scatter plot, Fig. 1(c), where the intensity of corresponding pixels is plotted with those from the *t* = 0 minutes image on the horizontal axis and those from the *t* = 5 minutes image on the vertical axis. Visually, image Fig. 1(b) is darker than Fig. 1(a) and higher pixel intensities from P_0_(x,y) tend to correspond with higher pixel intensities from *P_t_*_= 5_(*x*,*y*), although there is some variation from this. Quantitatively, this tendency is quantified by the correlation coefficient which, in Fig. 1(c), has been labelled as *r* = 0.77.

Therefore, this conveys that whilst the image intensity in Fig. 1(b) appears to have reduced from Fig. 1(a), the way that the pixel intensities vary is linearly related (*r* = 0.77), although not perfectly (*r* = 1.00). Therefore, correlation measures the linearity of the relationship between pixel intensities in two different images. A correlation coefficient of *r* = 0.00 indicates that the relationship is not linear.

Furthermore, having used *r* to determine the strength of the linearity, a straight line can be found that best describes the relationship between intensities at *t* = 0 and *t* = 5 minutes. The equation for such a straight line is given in Eq. (3), where the line is constrained to pass through the origin.

$$P_{t}(i)=bP_{0}(i)$$ (3)

In Eq. (3), *i* is an index variable enumerated over all pixels in each image. The slope *b* can be estimated by minimising the squared difference between the straight line and measured intensities, Eq. (4).

$$b(t)=\frac{\sum_{x}\sum_{y}P_{0}(x,y)P_{t}(x,y)}{\sum_{x}\sum_{y}P_{0}{\left(x,y\right)}^{2}}$$ (4)

Thus, the linear regression slope *b* provides a measure of the fractional intensity change. The correlation coefficient *r* provides a measure of association between the pixel intensities in two spatially registered, but temporally offset images. It quantifies how closely the intensities from the two images sit on the linear regression slope.

## 3. Materials and methods

### Sample preparation

**Fig. 2:**
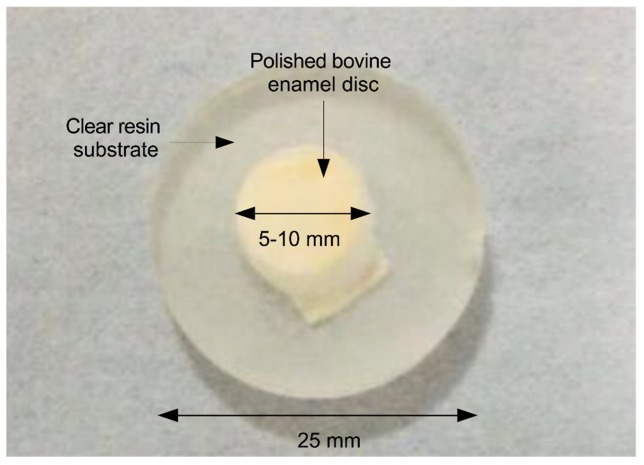
Bovine enamel disc measuring approximately 10 mm in diameter, embedded in a clear resin substrate having a diameter of 25 mm.

Sixteen bovine enamel samples from one-year-old calf incisors were sectioned into 5-10mm diameter discs, sliced and polished in the coronal plane to a thickness of 3 mm such that they comprised both dentine and enamel. The samples were then embedded into a 25mm diameter clear resin substrate as shown in Fig. 2

. The enamel surface was prepared by polishing to a flat specular finish, leaving a residual enamel thickness of approximately 0.5 mm on top of the underlying dentine. Preparation and mounting of the discs was undertaken by Modus Laboratories Ltd, Reading, UK. These specimen surfaces were painted with a thin layer of acid-resistant varnish in the form of nail polish, Revlon (570, New York, USA) to prevent erosion except on a 3x3 mm region. These samples were divided into two batches. The first batch of samples (n = 8) was used for micro-hardness measurements. The second batch of samples (n = 8) were imaged using OCT. Batching of the specimens into OCT and micro-hardness groups enabled the OCT measurements to be conducted without either specimen movement or drying, exploiting its non-invasive advantage. Micro-hardness measurements necessarily required specimen handling between each measurement.

### Acidic challenge

Artificial erosive lesions were induced by exposing the enamel specimens to an acidic solution of 1% citric acid (Sigma Aldrich, UK). The solution was buffered to pH 3.8 using Sodium Hydroxide (NaOH) pellets and stored at 25°C for a period of 2 hours prior to use. The pH of the solution was monitored throughout the experiments using a SenTix 41 electrode pH-meter (WTW GmbH, Germany).

**Fig. 3:**
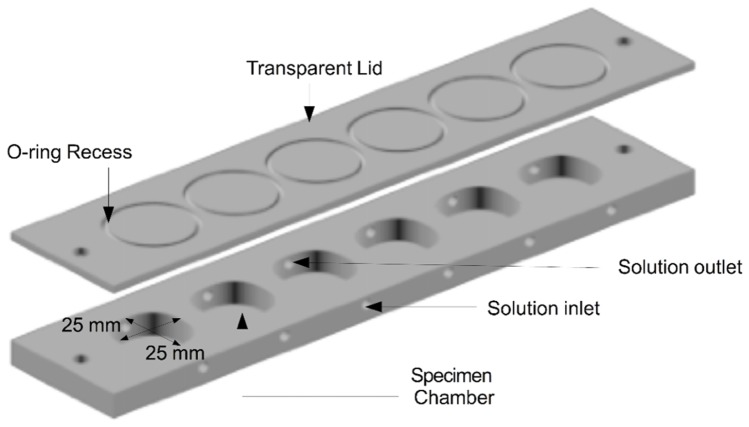
Specimen holder constructed from transparent PMMA and comprising 6 independent flow cell chambers. The total volume of each chamber was 2.3 mL.

A custom multiple specimen holder was used for this study. This comprised 6 isolated flow-cell chambers into which individual specimens were placed. The entire holder was constructed from transparent PMMA, comprising approximately cuboid chambers measuring 25x25x4 mm (width x height x depth) and having an actual volume of 2.3 mL, Fig. 3

(the true volume was slightly less than the cuboid volume due to rounded chamber edges). A single piece PMMA lid covered the compartments with each being isolated by a rubber O-ring. These were placed in semi-recessed grooves around each compartment such that a watertight seal was formed between the sample holder and its cover once screw fastened. The design of the chambers was such that they could accommodate approximately 0.8 mL of solution with the specimen and resin disc occupying the remaining volume (1.5 mL). The bovine samples were immersed in 100 ml of deionised water for 24 hours prior to exposure to the erosive agent. After the 24-hour hydration period, the samples were placed in isolated compartments within the sample holder, orientated with the enamel surface orthogonal to the OCT probe beam. A transparent cover was placed over the sample holder, leaving a 1 mm gap between the enamel surface and its inner surface.

The citric acid solution was introduced into each chamber of the holder through an inlet channel located below each specimen and ejected through an outlet channel located above. The solution was continuously refreshed at a rate of 5 mL/minute by a multi-channel pump (323Du/D, Watson Marlow, UK), drawing fresh solution from a 1 L reservoir and ejecting used solution into a separate container. Both the inlet and outlet channels were connected to different channels of the pump using silicone tubing and plastic connectors. The tubing was connected to the chamber inlet and outlet channels using winged 21 gauge syringes.

Experimentally, the specimen holder was mounted vertically such that the enamel surface was perpendicular to the bench. This configuration was chosen following experiments whereby bubbles formed on the enamel surface and prevented clear OCT images from being acquired. The vertical orientation mitigated against this, allowing the bubbles to rise away from the field of view. However, by operating in this regime, the bottom of the specimen was exposed to the acid challenge a few seconds before the top. Filling of the sample compartments with 0.8 mL solution took less than 10 seconds. Therefore, this was assumed to have a negligible impact upon the results. Once all sample holders were full, OCT imaging commenced.

### OCT system specification and configuration

The OCT system used for this study was custom designed and built in-house. The instrument incorporated a fibre optic Mach-Zender interferometer design utilising a super-luminescent light emitting diode (SLED) optical source (SLD1325, Thorlabs, Cambridge, UK), operating with a nominal central wavelength of 1325 nm and bandwidth of approximately 100 nm. The axial and transverse resolutions were measured to be approximately 8 μm and 10 μm in air respectively.

Interference fringes were detected at an A-Scan rate of 50 kHz, comprising an InGaAs linear detector array (SU-LDH2, Sensors Unlimited, USA), a reflective diffraction grating and focusing/collimating achromatic doublet lenses. Images were obtained by raster scanning the probe beam over the tissue surface using a two-dimensional galvanometer configuration (GVS012, Thorlabs, UK). The light was focused approximately 100 µm beneath the sample surface by a scan lens (LSM03, Thorlabs, UK). The system acquired, processed and displayed 3D OCT data in real-time using a General Purpose Graphics Processing Unit (NVIDIA C2070) [32]. Three-dimensional OCT volumes were acquired as a series of 500 B-Scan images, each comprising 500 A-Scans, of length 512 pixels. Physically, the lateral pixel spacing between B-Scans and A-Scans was 6.9 μm and the axial pixel spacing was measured to be 9.5 μm in air. The group refractive index of sound enamel is approximately 1.65 [33], yielding an approximate axial pixel spacing of 5.7 μm within the sample. The system point-spread function and geometric scaling were measured using the method and phantoms described previously [34–36].

**Fig. 4:**
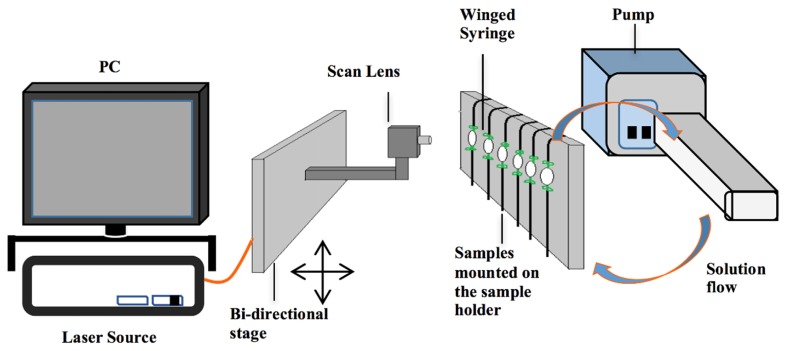
Experimental setup used to capture OCT images during the development of early stage surface softening in bovine enamel. The OCT probe was mounted on a linear translation stage to enable imaging of multiple specimens. The specimens were mounted vertically in a flow-cell through which acidic solution was pumped.

The OCT system was configured to automatically measure multiple specimens by mounting the OCT imaging probe onto a motorised linear translation stage (LTS300, Thorlabs, UK). The translation stage repositioning accuracy was 2.0 ± 1.7 μm. Acquisition of a single OCT volume and translation between specimens took no more than 20 seconds, enabling OCT image volumes of three different specimens to be imaged at up to once per minute. The experimental configuration is shown in Fig. 4

.

### OCT measurement protocol

Prior to introducing the acid challenge, the OCT imaging system was aligned such that the top of each specimen was 50 pixels from the top of each B-Scan and the *en face* field of view was adjusted to ensure that a section of the varnished surface was included in each volume as a reference zone. This alignment procedure required that the specimen chambers were filled with deionised water and facilitated the acquisition of an initial baseline OCT volume for each sample. Following alignment, the deionised water was pumped from the specimen chambers leaving the alignment intact.

Three-dimensional OCT image volumes were acquired from each sample over a square surface area measuring 3x3 mm corresponding to the unvarnished window. To ensure image acquisition of the first few minutes of erosion, the system was set to acquire the first 20 volumes into computer memory at an average rate of one volume every 10 seconds. Once this buffer was full, the subsequent volume imaging rate was limited to 22 OCT volumes over a 2 hour period. This procedure ensured that all specimens had been imaged twice within the first 2 minutes of the acid challenge.

OCT experiments were repeated four times. In the first and second experiments, erosion was measured in 3 bovine enamel specimens, moving the OCT measuring probe between specimens as described above. The third and fourth experiments measured single specimens eliminating the need to move the OCT probe between acquisitions.

In the first and second experiments, a scattering reference phantom was measured in its own specimen chamber. The reference phantom comprised a suspensions of Titanium Dioxide powder embedded within a polyurethane matrix with a mass ratio of 6.11 mg g^−1^, resulting in a scattering coefficient of 3.5 mm^−1^ [37]. This was measured automatically before each cycle of specimen measurements, providing a constant baseline for reference. This was preferable to measuring an enamel specimen which would be expected to undergo some partial demineralisation in the de-ionised water which was under-saturated with respect to hydroxyapatite.

### OCT data processing and correlation analysis

Integrated *en face* projections were generated from each 3D OCT data set by selecting all C-Scans from the surface to a depth of approximately *z*_max_ = 500 μm and summing the pixel intensities together to give a single composite image. The depth of 500 μm corresponds with the Rayleigh range of the OCT objective and was chosen to ensure that the ESS region was always within the axial range contributing the highest OCT signal. Furthermore, it was observed that the sections of the bovine enamel were nominally 500 μm thick.

Assuming the *en-face* surface view (C-Scan) to be in the *x*,*y* plane and the orthogonal axial direction to be in *z*, then the projected image *P*(*t*,*x*,*y*) acquired at time *t* is given by Eq. (5)

$$P(t,x,y)=\sum_{z=z_{\text{surface}}}^{z_{\text{max}}}I(t,x,y,z)$$ (5)

where *I(t*,*x*,*y*,*z)* represents the volumetric OCT data. Notably, the OCT volumes used in this study were linearly scaled intensities. Thus, these are not the same as post-processed OCT volumes that are typically comprised from a stack of logarithmically scaled B-Scan images.

**Fig. 5:**
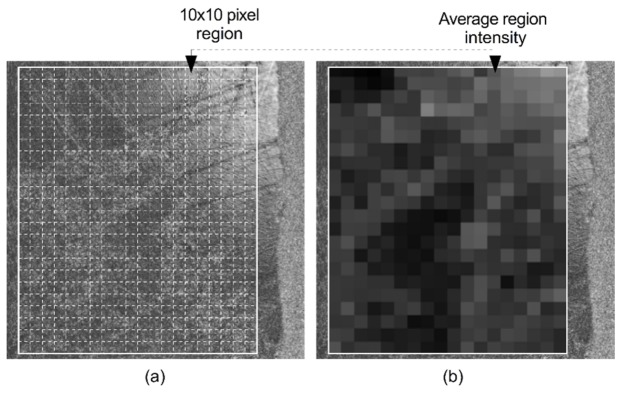
Sub-sampling of an OCT surface projection image. a) The original projection image overlaid by a region of interest (ROI) comprising a grid of 10x10 pixel regions. b) Each 10x10 pixel region replaced by its corresponding mean pixel intensity.

From the projection image, a region of interest (ROI), measuring (0.65x0.90 mm) was manually selected for each sample to avoid the varnished sections of enamel and intense Fresnel reflections that saturated the OCT spectrometer. To mitigate against small measurement-measurement movements, the ROI was sub-sampled on a grid of 10x10 pixel regions, replacing the pixel intensities by the mean for that region. This is shown schematically in Fig. 5

.

Thus, the correlation *r* and regression slope *b* were calculated using the mean region intensities rather than individual pixels. ROI selection and cropping was carried out using ImageJ [38] and calculation of the correlation value and regression slope was achieved using the CorrelationJ plugin [39] and MATLAB.

### Surface microhardness measurement protocol

A Vickers micro-indenter (HMV Microhardness Tester, Shimadzu, Japan) was used to measure the enamel surface hardness. Prior to acid challenge, a control region of the enamel surface from each specimen was masked using nail varnish to prevent erosion.

A batch of fresh citric acid (1% w/v) at pH 3.8 was prepared and measured into 100 ml cylindrical glass containers, one for each specimen. Each specimen was submerged in fresh solution, exposing it to an acid challenge for a pre-determined time before being removed and washed thoroughly with de-ionised water. Specimens were left to dry for 5 minutes at room temperature prior to hardness measurement. Hardness measurements were made following cumulative acid challenge durations of 5, 10, 15, 30, 45 and 60 minutes. The solution was refreshed after each cycle of erosion.

Once dry, the enamel block surface was placed flat on the translation stage, perpendicular to the indenter direction of load. A central area of approximately 1 mm × 1 mm was identified for hardness testing. Micro-indents were made with a load of 200 g applied for 10 s. The mean of six separate indentations, approximately 100 μm apart, was calculated for each sample. Vickers hardness numbers were calculated for each indentation and averaged for each specimen and time-point.

## 4. Results

**Fig. 6:**
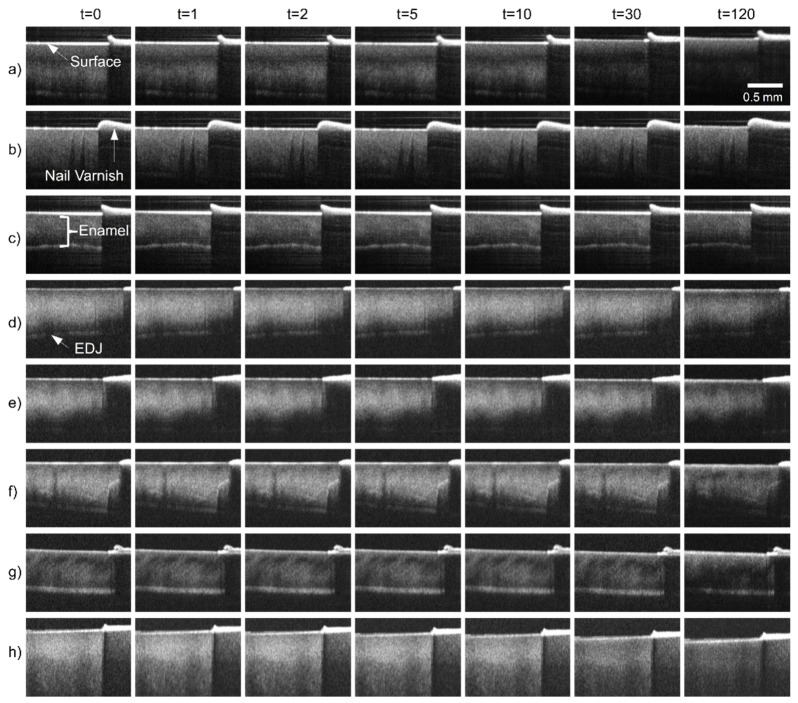
OCT B-Scans (logarithmic intensity) taken from the center of each specimen at time-points, t = 0, 1, 2, 5, 10, 30 and 120 minutes. Rows a) to c) correspond to the first experiment, d) to g) the second and h) the third experiment. The images show the enamel surface, nail varnished region, enamel and enamel dentine junction (EDJ).

Centre B-Scans from each of the measured specimens are shown in Fig. 6(a)-6(h)

at time-points *t* = 0, 1, 2, 5, 10, 30 and 120 minutes of continuous acid challenge. The B-Scans represent cross-sections of the bovine enamel discs, showing the surface, bulk enamel and nail varnish used to mask regions of enamel from the acid challenge. In addition, the EDJ is visible in specimens a)-g). In image Fig. 6(h), there is a distinct intensity change approximately halfway along the axial dimension. It is possible that this represents the EDJ, however the appearance is not consistent with specimens a)-g). Direct comparison of OCT B-Scan images obtained at different times was facilitated by acquiring the OCT data at the same lateral location within each specimen, limited by the positional repeatability of the linear translation stages. However, axial displacement of up to approximately 100 µm is evident in specimens (a), (g) and (h).

**Fig. 7:**
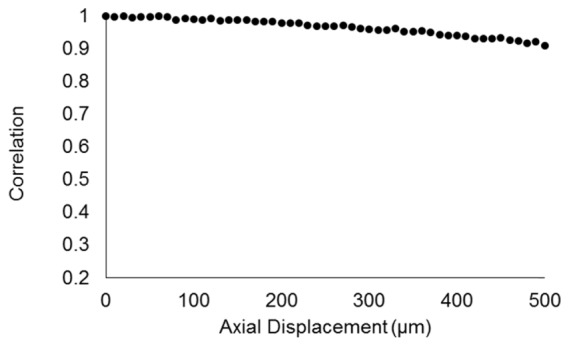
Correlation coefficient measured for an enamel specimen displaced axially in 10 µm increments over a total displacement of 500 µm.

The potential impact of axial displacement on the correlation measurements was assessed at 10 µm axial increments over a total displacement of 500 µm. The results are shown in Fig. 7

, revealing a decrease in correlation to *r* = 0.91. However, within 120 µm of axial displacement the correlation dropped to no less than *r* = 0.98. Therefore, no additional processing was undertaken to account for axial displacement.

Despite steps taken to physically normalize the specimens (i.e. polishing, enamel type and source), each specimen had a distinct morphological appearance when imaged by the OCT instrument. In particular there is a variation in the intensity of the images. Nevertheless, some visual consistencies are observed. Firstly, over the duration of the acid challenge, the B-Scan image intensity decreases. However, the B-Scan intensities are logarithmic, leading to compression of intensity variations. Over the initial 10 minute period, no visual changes to the surface are apparent in the B-Scans. Such changes become visible by 30 minutes. However, comparing the enamel surface to the nail varnish, there is no discernible axial loss of enamel.

**Fig. 8:**
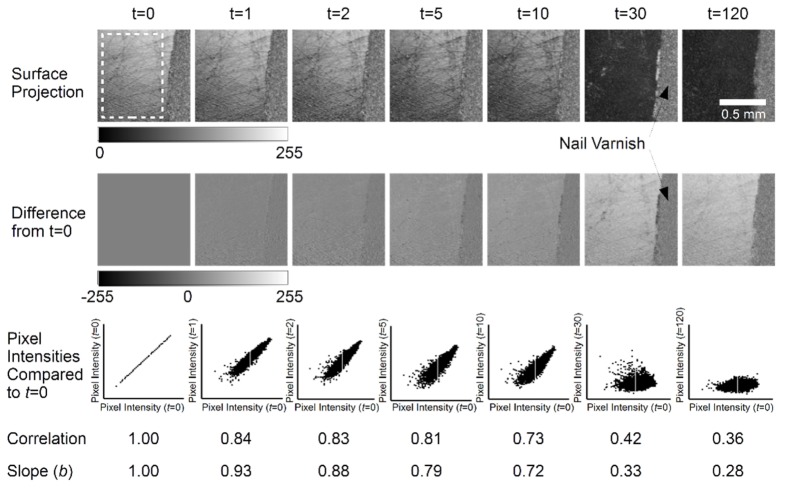
Results for a single specimen from the first experiment at t = 0 (baseline) and subsequent time-points t = 1, 2, 5, 10, 30 and 120 minutes. Surface projection images are calculated from linearly intensity OCT volumes as described in the text. The difference images represent the projection image difference from baseline. The pixel intensity scatter-plots compare the mean intensity of corresponding 10x10 pixel regions in base-line and subsequent images. Correlation values are calculated from each scatter-plot.

**Fig. 9:**
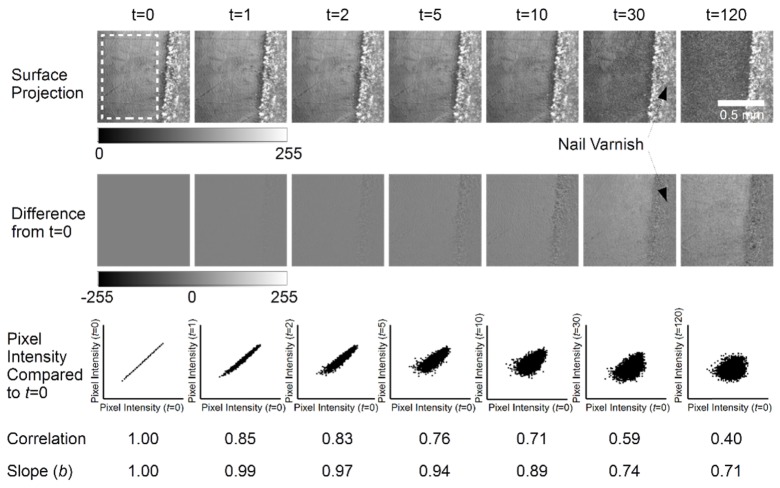
Results for a single specimen from the second experiment at t = 0 (baseline) and subsequent time-points t = 1, 2, 5, 10, 30 and 120 minutes. Surface projection images are calculated from linearly intensity OCT volumes as described in the text. The difference images represent the projection image difference from baseline. The pixel intensity scatter-plots compare the mean intensity of corresponding 10x10 pixel regions in base-line and subsequent images. Correlation values are calculated from each scatter-plot.

The cross-sectional B-Scan view of the 3D OCT volumes is limited in its ability to convey information about ESS, which is necessarily a surface phenomenon. Therefore, projection images were computed for each specimen at all measured time-points from their corresponding 3D OCT data sets. The difference between baseline (*P*_0_) and a subsequent projection image (*P*_t_) acquired at time *t*, d*P* = *P*_0_-*P*_t_, were calculated in order to facilitate visualisation of changes due to the acid challenge. Pixel region intensity scatter-plots were produced for each specimen between the baseline and subsequent images and the corresponding correlation coefficient was calculated. Results for *t* = 0, 1, 2, 5, 10, 30 and 120 minutes are shown for specimens from the first (Fig. 8

) and second (Fig. 9

) repeats of the experiment. The white dashed region in the baseline projection image at *t* = 0 indicates the area selected for correlation analysis.

The projection images (top row) show a superposition of the linear OCT intensity signal backscattered from the specimen surface to a depth of approximately 500 µm below the surface. However, the surface signal dominates due to an approximately exponential signal attenuation axially.

The results from the first specimen, Fig. 8, show some visually subtle darkening of the projection images over the first 10 minutes of the acid challenge, which becomes pronounced after 30 minutes. By 120 minutes there is little visual difference from the projection image at 30 minutes. These changes are reflected in the difference images which show a progressive intensity difference from baseline. After the first 10 minutes of acid challenge the correlation was found to be *r* = 0.73, compared to a theoretical maximum of *r* = 1.00 at baseline. By 120 minutes the correlation had dropped to *r* = 0.36. The nail varnish remained relatively unchanged throughout. The projection image shows surface scratches from the polishing process, these remain evident at 10 minutes, although they are not visible at 30 minutes. The pixel intensity slope shows that the projection image pixel regions at 10 minutes are darker by *b* = 0.72 times the baseline pixel regions, i.e. its intensity is 72% that at baseline. This is visually perceivable. However, darkening at *t* = 1, 2 and 5 minutes is more subtle, but detectable by pixel intensity slope values of *b* = 0.93, 0.88 and 0.79 respectively.

Similarly, the second specimen, Fig. 9, shows a consistent projection signal from the varnished region and a progressive intensity decrease. Surface scratches are still evident, however, they appear less prominent than in the first specimen. The temporal intensity decrease appears less than observed in the first specimen, with visual intensity changes up to *t* = 10 minutes not clearly visible. Furthermore, whilst it is difficult to subjectively discern an intensity change within the first 5 minutes, objectively the slope reduces as *b* = 1.00, 0.98, 0.97, 0.93, 0.89 at corresponding time-points of *t* = 0, 1, 2, 5 and 10 minutes. Thus, the projection image at *t* = 10 minutes is approximately 89% the intensity of the baseline image. Compared with the specimen shown in Fig. 8, the projection exhibits a smaller intensity change over the 120 minute acid challenge, i.e. 71% compared to 28% of baseline intensity. Notably, the correlation values drop from *r* = 1.00 to *r* = 0.71 in the initial 10 minute period of acid challenge.

**Fig. 10:**
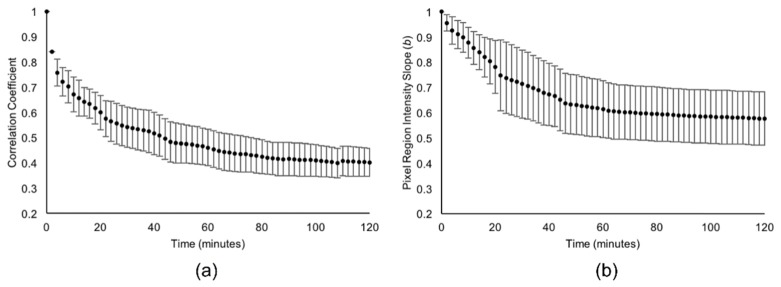
Mean (a) correlation coefficient and (b) image intensity slope at 2 minute intervals measured over a continuous 120 minute erosive challenge. The mean is calculated over all 8 specimens. The error bars represent 95% confidence intervals.

Each specimen was measured by OCT sequentially. Therefore, there was a necessary time delay between measurement of each specimen. In order to calculate the mean correlation and slope parameters, the respective curves for each specimen were linearly interpolated. The mean correlation and slope values and their 95% confidence intervals were calculated at 2 minute intervals and plotted in Fig. 10(a)

and Fig. 10(b) respectively.

The mean correlation coefficient exhibits a smooth decrease from *r* = 1.00 at baseline to *r* = 0.40 ± 0.05 at *t* = 120 minutes. Similarly, the mean pixel region intensity slope decreases over time from b = 1.00 at baseline to b = 0.58 ± 0.11 after 120 minutes, indicating that on average, after 120 minutes of acid challenge, the projection images darken to 58% of their original intensity. Furthermore, the linear correspondence between pixel region intensities in the images at the two time points accounts for *r*^2^ = 16% of the intensity variation. However, the correlation values show three times less sample-to-sample variation, with a median variance of 0.03 compared to 0.09 for the slope measurements. The difference was statistically significant (*p*<<0.05, Mann-Whitney U Test), indicating that the correlation was a more repeatable measure.

**Fig. 11:**
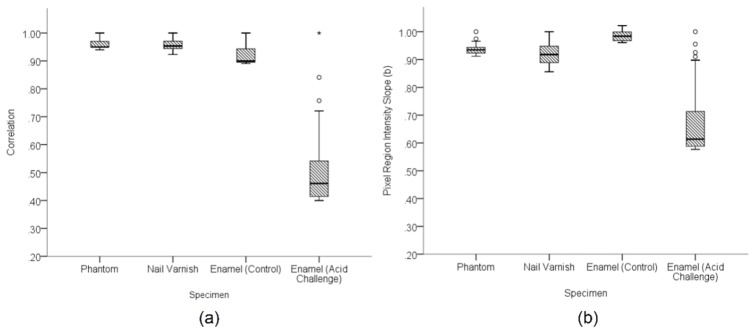
Comparison of the correlation range for the reference phantom, nail varnish, bovine enamel immersed in de-ionised water and bovine enamel immersed in acidic solution. Measurements were obtained over a 120 minute continuous immersion period.

The decreasing correlation values observed in enamel specimens throughout acid challenge were compared to results from a static polyurethane scattering phantom, the nail varnished region and a control enamel specimen. The latter of these was immersed only in de-ionised water. All were measured under the same conditions for a period of 120 minutes. The variation of the resulting correlation and slope values were plotted as Box and Whisker Diagrams, Fig. 11(a)

and Fig. 11(b) respectively. The Box plots show minimum correlation values for the phantom, nail varnish and enamel control of r = 0.95, r = 0.92 and r = 0.89 respectively. The minimum enamel correlation value obtained under acid challenge was r = 0.40.

One-way analysis of variance (ANOVA) and Tukey’s Honest Significant Difference (HSD) Test were used to determine the statistical similarity between the control specimens (phantom, nail varnish and enamel in water). This revealed no statistically significant difference between the phantom and nail varnish correlation values (*p =* 0.93). However, the enamel exposed only to de-ionised water for 120 minutes yielded significantly different correlation values from both (*p*<0.05). The phantom pixel intensity slope was statistically similar to both the nail varnish (*p* = 0.10) and the enamel (*p* = 0.13) at the 5% significance threshold. However, the nail varnish and enamel were significantly different (*p<<*0.05).

**Fig. 12:**
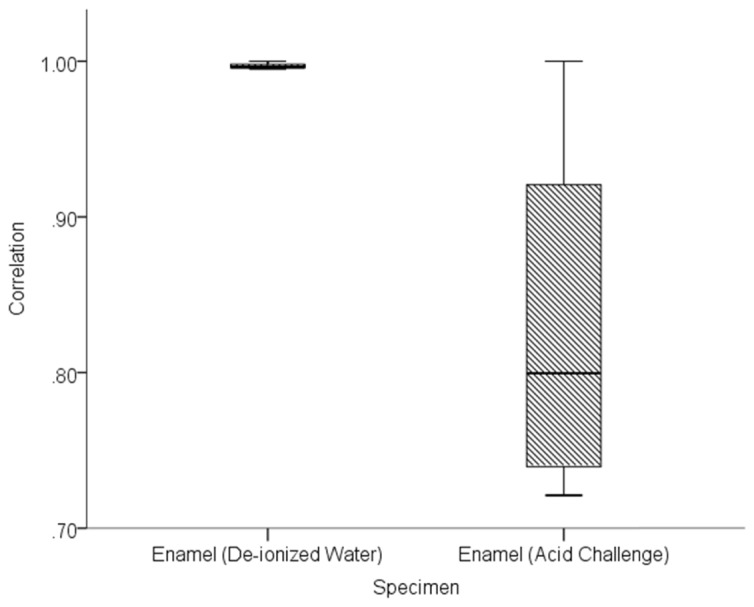
Comparison of correlation values obtained at one minute intervals during 5 minutes immersion of bovine enamel in de-ionised water and citric acid at pH 3.8.

Of particular interest in this study, was the sensitivity of OCT to enamel changes occurring within the first few minutes of acid challenge. Figure 12

visually compares correlation values obtained from two enamel specimens immersed in de-ionised water and citric acid (pH 3.8) for 5 minutes.

Five minutes immersion of bovine enamel in de-ionised water resulted in a correlation range of Δ*r* = 0.01 and median *r* = 0.99. This compares with a correlation range of Δ*r* = 0.28 and median *r* = 0.80 obtained from bovine enamel during a 5 minute acid challenge.

**Fig. 13:**
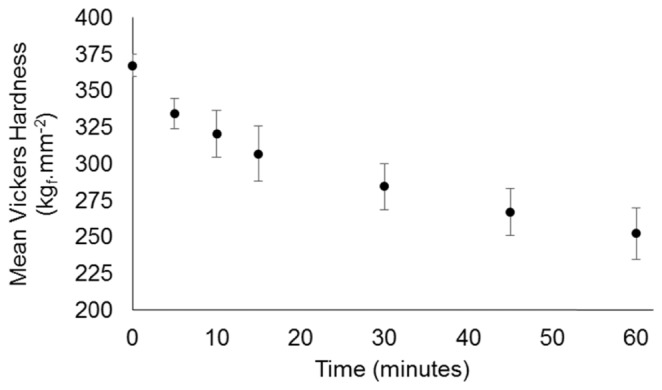
Mean Vickers hardness measurements acquired from a batch of 8 bovine enamel discs each subjected to a total of 1 hour of acid challenge and measured after 0, 5, 10, 15, 30, 45 and 60 minutes.

In order to assign physical meaning to the correlation and slope parameters, an independent batch of bovine enamel specimens were subjected to acid challenge and micro-hardness analysis. The mean Vickers hardness (HV) was calculated from all of the measured specimens. The results are plotted in Fig. 13

, with error bars representing 95% confidence intervals.

**Fig. 14:**
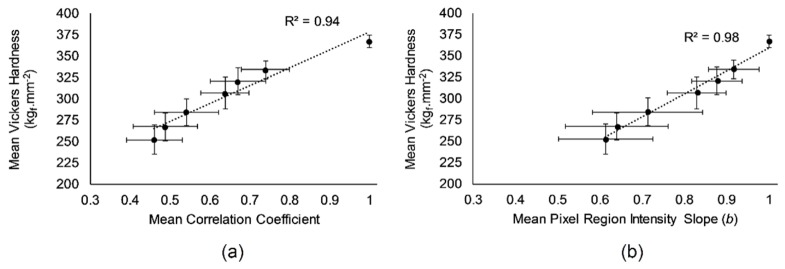
Mean Vickers hardness measurements for 8 bovine enamel specimens following 0, 5, 10, 15, 30, 45 and 60 minutes of acid challenge plotted with the corresponding correlation values (a) and pixel region intensity slope values (b). Error bars represent 95% confidence intervals. A linear regression line is plotted in both.

The mean Vickers Hardness ranged from 367.1 ± 14.1 kg_f ·_mm^2^ at baseline to 252.4 ± 32.8 kg_f ·_mm^2^ after 60 minutes of erosive challenge. The average micro hardness profile, shown in Fig. 13, follows a visually similar trend to that of the correlation and intensity slope curves shown in Fig. 10(a)-10(b). This relationship is shown in Fig. 14

by plotting the correlation coefficients (a) and slope parameter (b) from OCT as a function of measured surface hardness for the two independent batches of specimens.

A linear regression of surface hardness as a function of measured correlation yielded a quantitative relationship between the OCT measurements and surface hardness. The slope parameter demonstrated a higher goodness of fit with *r*^2^ = 98%, compared with the correlation parameter having *r*^2^ = 94%. Thus, on average, the regression lines suggest that for the experimental conditions employed here, correlation values were related to the surface hardness measurements by the expression HV = 209.8*r* + 168.6. Likewise, pixel region mean intensity slope values were related to hardness by HV = 269.0*b* + 89.9.

## 5. Discussion

The set of experiments reported in this paper aimed to demonstrate an OCT based measurement and analysis protocol capable of measuring the formation of ESS and erosive lesions prior to the net loss of enamel that was not directly observable in OCT as an axial change. Following 120 minutes of continuous acid challenge (pH 3.8, citric acid) the net loss of surface enamel was expected to be nominally 10 µm, corresponding to approximately 1-2 axial pixels in the OCT image. The OCT B-Scans in Fig. 6 support this level of erosion, showing little sign of enamel surface loss relative to the nail varnished surface. Therefore, the net enamel loss appeared to be at the limit of what was directly measureable from images, using basic measuring techniques such as ImageJ’s line tool. However, more sophisticated techniques such as surface peak fitting or phase sensitive methods could potentially detect substantially smaller changes at the nanometer scale [40]. Nevertheless, such techniques are sensitive to temporal stability of the specimen and experimental setup and it remains to be shown whether they are sensitive to ESS.

In this work, the specimens were monitored by OCT under continuous acid challenge, without moving or disturbing the samples. In addition to this, three-dimensional volumetric images of the samples were acquired at each measurement. These aspects are important because they ensure that the same enamel locations were longitudinally compared, facilitating assessment of the surface. ESS and erosive lesion development are fundamentally surface phenomena. This is highlighted by the B-Scans in Fig. 6, which show no visible morphological change over time, except to the few rows of pixels corresponding to the surface. These were only visibly modified after 30 minutes of acid challenge. In addition to this, B-Scan visualisation between teeth was not consistent, varying between samples. This is likely due to inter-sample variation, an inherent issue with biological specimens. The formation of surface projection images transforms the OCT data into a more appropriate form for surface analysis. In this rendering, the projections visibly show the surface (i.e. Fig. 8 and Fig. 9) and the change in back-scattered intensity. Changes to OCT B-Scan intensity under demineralising conditions have been previously studied. In this work, a new approach to intensity variations was used. The mean of local pixel intensities were compared between each time-point and baseline, by regressing a straight line through the origin using Eq. (3). The slope of this line describes the fractional intensity of each projection image in terms of the pixel region intensities at baseline. By formulating the data in this way, it is evident that the regression slope contains only part of the available information. The strength of the relationship, or tendency of the pixel intensities to sit on the straight line, is described quantitatively by Pearson’s product moment correlation coefficient. Therefore, the correlation coefficient measures how similar projection images are, regardless of intensity scaling. This fact is readily supported by considering that the correlation coefficient of any set of points along a straight line is equal to unity regardless of the slope.

The correlation coefficient (Fig. 10(a)) and slope (Fig. 10(b)) were both observed to decrease with acid challenge time. This change was attributed to the effect of acid challenge because it substantially differed from the three control specimens; a polyurethane phantom, nail varnished region and bovine enamel immersed in de-ionised water. The comparisons between these controls and both the correlation and slope parameters due to acid challenge were shown in Fig. 11(a) and Fig. 11(b) respectively. There were also some smaller differences amongst the controls. Notably, the enamel in de-ionised water exhibited a different correlation range to the phantom and nail varnish. This is perhaps to be expected because the lack of ions in the solution necessarily induces some demineralisation during the 120 minute immersion period. Arguably Fig. 11 shows that OCT correlation was sensitive to these changes. The slope parameter for the control enamel showed little change, indicating the overall intensity of the specimen remained largely unchanged. However, the slope parameter for the nail varnish was observed to decrease, agreeing with experimental observations of a visual change in appearance, possibly due to some water absorption into the varnish surface. Correlation for the nail varnish remained unchanged over time, statistically similar to the phantom. This is consistent with the varnish morphology remaining intact, despite some possible water absorption modifying the intensity of backscattering. Thus, both the phantom and nail varnish appear to be suitable reference specimens for longitudinal correlation.

The correlation and intensity slope parameters followed an approximately exponential decay as a function of acid challenge duration. This was seen also in hardness measurements, whereby the initially rapid softening has been explained by an immediate dissolution of mineral from the surface, thus partially exposing deeper enamel to acid challenge and subsequent partial dissolution. This process repeats, eventually forming a sub-surface lesion that is just evident in the OCT B-Scans (Fig. 6) at *t* = 120 minutes. However, dissolution of the initial enamel surface continues, resulting in net erosion. Thus, a sub-surface softened region is constantly formed at depth below the eroding surface. The result is a hardness curve of the form shown in Fig. 13. The similar form of both the slope and correlation curves can likewise be explained by the combination of surface and sub-surface changes occurring. Initially, changes to the projection images are dominated by the specimen surface. However, once the surface softening is established, sub-surface demineralisation is expected to dominate the projection images. However, the exact mechanism by which chemical and morphological changes affect the OCT measurements remains to be elucidated. Nevertheless, this study has shown that OCT derived measurements are quantitatively sensitive to ESS. Furthermore, correlation between local pixel region intensities was found to be a statistically more reproducible measure of ESS than the intensity slope. This is evidenced by the tighter error bars in Fig. 10(a) compared to Fig. 10(b) indicating less inter-specimen variation for correlation measurements. However, up to 20 minutes acid challenge, the error bars also indicate comparable variation in the slope parameter compared to correlation. This makes sense because demineralisation is a cumulative process. The OCT measurements reference to the specimen at *t* = 0 and therefore, at early time-points the variation between specimens is necessarily limited, i.e. there is a distribution of specimen susceptibility to demineralisation. However, the cumulative effect compounds the variation such that it increases with time until an equilibrium is reached. Therefore, once demineralisation is established, the rate of change of OCT projection image intensity tends towards a constant. This theory is supported by the linear relationships with surface hardness.

The hardness measurements were obtained from a separate batch of bovine samples to the OCT measurements. This was necessary because the aim of the OCT measurements was to measure *in situ* without disturbing the specimens. Conversely, hardness testing necessarily required specimen handling and preparation. Thus, the correlation and slope relationships shown in Fig. 14(a) and Fig. 14(b) are statistical, relying upon the measurement of multiple different specimens to represent true underlying sample variation sufficiently to show an average relationship between the two experiments. The results indicate a strong linear relationship (*r*^2^ = 94% and 98%, *p* << 0.05) and thus support the idea that both backscattered intensity and projection image similarity can be used as non-invasive proxies for hardness, notably this appears to hold within the first 10 minutes of ESS formation thus providing a way to monitor early lesion formation without disturbing the specimen. The quantitative relationships determined from the regression lines between correlation/ slope and hardness are necessarily empirical. Therefore, careful calibration would be required in order to use OCT as an optical hardness measuring system. However, these results show the potential for such use.

In this work, the projection images were sub-divided into 10x10 pixel regions, the mean of each being used for all subsequent calculations. The purpose of this was to mitigate against any lateral re-positioning uncertainty that might occur due to imaging multiple specimens and movement of the OCT probe on a linear translation stage. Region sizes were tested from 3x3 to 10x10 pixels. The results of this testing have not been included here, however, the outcome of such tests revealed negligible sensitivity to the region size.

Projection images were formed over a depth of 500 μm, one hundred times the expected depth of the softened layer found in ESS. The rationale for this is as follows. Erosive changes predominantly effect the enamel surface to a depth of 2-5 μm. When the initial surface is polished, as it is here, the initial change is from a specular reflection to a slightly diffuse scattering interaction. Thus, the intensity of light coupled back into the OCT collection optics immediately from the surface is reduced. Furthermore, the intensity of light transmitted into the specimen without interaction and backscattered from within the sample back through the diffuse surface is also modified. The net result of this change is a reduction in the B-Scan image intensity, this is visually apparent in Fig. 6. Whilst it has been shown to be possible to detect erosive changes by concentrating on the intensity of only the surface reflection [20,27], these approaches ignore the integrated effect at depth that is more than simply a shadowing influence of the surface. When considering this effect, it is important to remember that OCT B-Scan images are typically rendered on a logarithmic scale. In linear units, the axial OCT signal corresponding to a homogeneous scattering medium is typically represented by a strong initial surface reflection peak followed by an exponential intensity attenuation. Thus, integrating the OCT signal over some axial depth is akin to performing an exponential weighted average, with preference for the surface signal. This is expected to increase the sensitivity to subtle surface changes by incorporating the effect of these changes from sub-surface regions. This study has not attempted to optimise the projection depth. Instead, the Rayleigh range of the objective was chosen as a reasonable estimate over which the collection efficiency is relatively uniform. Further work is required to assess the impact of projection depth on the overall results. However, this work has explicitly integrated over the OCT linear intensity signal that is available from the raw data acquired by the custom OCT system used for this study. Previous studies have utilised the post-processed B-Scan data that is on a logarithmic scale.

The results, protocol and experimental setup described in this paper have important implications. For example, ESS is considered to be a reversible change because an enamel framework remains, into which new mineral can be deposited. Therefore, by adopting this setup, not only can ESS formation be monitored non-invasively, but the potential exists by which therapeutic agents could be introduced into the solution and the effects monitored, all without disturbing the samples. Furthermore, should it be necessary to physically apply a treatment to the enamel surface, the specimen holder can be drained and opened without disturbing the specimen position. Thus, spatially registered measurements can be made to assess new interventions.

This work has demonstrated that pixel region correlation of 4D OCT data can optically detect ESS formation in bovine enamel. Further work is now required to establish how OCT data acquired in this way can be used to measure changes that occur when such lesions are remineralised.
